# Comparison of Gut Viral Communities in Atopic Dermatitis and Healthy Children

**DOI:** 10.3389/fmed.2022.835467

**Published:** 2022-02-21

**Authors:** Xiang Lu, Hao Wang, Jingqi Zhang, Kexin Jin, Ling Ma, Yan Wang, Shixing Yang, Xiaochun Wang, Quan Shen, Tianji Zhou, Hui Xu, Wen Zhang

**Affiliations:** ^1^Department of Dermatology, The Affiliated Hospital of Jiangsu University, Zhenjiang, China; ^2^Department of Microbiology, School of Medicine, Jiangsu University, Zhenjiang, China; ^3^Department of Clinical Laboratory, The Affiliated Huai'an Hospital of Xuzhou Medical University, Huai'an, China; ^4^School of Mathematical Sciences, Soochow University, Suzhou, China

**Keywords:** atopic dermatitis, virome, gut, metagenomics, diversity

## Abstract

The immune development and regulation of living individuals are affected by the gut microbiota. The imbalance of gut microbiota is considered to be a key factor that easily induces immune dysregulation and the development of atopic diseases. Atopic dermatitis (AD) is a chronic inflammatory skin disease that affects nearly 20% of children. To date, metagenomics research on AD has mainly focused on the skin and gut microbiome. However, here we assessed the composition of the virome in the gut of AD patients and healthy controls for the first time. This study has obtained possible dominant viruses at different viral classification levels. In terms of diversity, the alpha diversity of the patients group was significantly lower than that of the healthy controls group, and the beta diversity of the two groups was significantly different from phylum to family level. These findings provide a new perspective for us to better understand the effect of the gut microecological environment on AD.

## Introduction

In the past few decades, the prevalence of allergic diseases in developed and developing countries has increased disproportionately (1). Atopic dermatitis (AD) is the most common recurrent chronic inflammatory skin disease with a genetic predisposition, affecting approximately 20% of children worldwide, of which 45% develop in the first 6 months of life (2). About half of children with moderate or severe AD can develop allergic rhinitis with or without asthma, and other atopic diseases with low mortality (3). The “hygiene hypothesis” put forward in the late 1980s holds that environmental and nutritional factors may cause ecological disorders of the skin, gut, or lung microbiome (4, 5). The microbiome of these sites can regulate the immune response and reduce the stimulation of the immune system by microorganisms, making infants more susceptible to allergic diseases. Although the mechanism driving the outbreak of AD is not fully understood, and its severity is highly heterogeneous, skin barrier dysfunction, microbial skin colonization, decreased innate immune responses, and other external factors may have a complicated interrelationship with AD (6).

The role of microbiome in AD has always been concerned. It has been reported that there are significant differences in microbial alpha and/or beta diversity and abundance between patients with AD and healthy people (7, 8), and proportion of *Staphylococcus aureus* sequences detected in the skin of AD patients is related to the severity of the disease (3). However, since the skin is directly affected by the external environment, samples collected from the skin are more susceptible to contamination than samples collected from inside the body. The gut microbiome is considered to be closely related to human health and participates in human metabolism, food digestion and utilization, and immune response. Several studies have shown that infants with AD have different microbiome composition from healthy controls (9–11). In addition, a recent report found that compared with the healthy controls group, *Sutterella, Faecalibacterium, Bacteroides*, and *Parabacteroides* are increased in the gut microbiome of AD children, while *Eubacterium, Blautia, Propionibacterium*, and *Bifidobacterium* are reduced (5). It has been reported that, in individuals with certain disease states (e.g., obesity and type 2 diabetes), the composition of gut virome (particularly phages) is closely related to the composition of bacteria (12). Therefore, it is necessary to explore whether there are differences between gut virome in AD patients and healthy individuals.

In this study, we further investigated the composition and differences of the gut viral community between 21 children with AD and 12 healthy children, which provided new insights for us to better understand the impact of gut microecological environment on AD.

## Materials and Methods

### Sample Collection and Preparation

From September 2017 to August 2018, sterile disposable cotton swabs were used to collect stool samples from 21 AD patients admitted to the Department of Dermatology, Affiliated Hospital of Jiangsu University and 12 healthy controls, approximately 10 g of feces were taken from each sample. All AD patients fulfilled the diagnosis criteria of Hanifin and Rajka. All specimens were stored in sterilized covered containers and transported over dry ice. Prior to viral metagenomic analysis, the tips of collected swabs were immersed in 1 ml Dulbecco's phosphate-cushioned saline (DPBS) and vortexed for 5 min vigorously, then incubated at 4°C for 30 min. After centrifugation (10 min, 15,000 g), the supernatants were collected in 1.5 ml centrifuge tubes and stored at −80°C for later use (13).

### Viral Metagenomic Analysis

About 500 μl of supernatant was drawn from each sample and collected into the separate sample pool. All samples were centrifuged at 4°C for 5 min at 12,000 g in order to remove bacterial cell-sized and eukaryotic particles and filtered through a 0.45 μm filter (Merck Millipore, MA, USA) to enrich virus particles protected from digestion by viral capsids. Filtrates were then treated with DNase (Turbo DNase from Ambion, Thermo Fisher, Waltham, MA, USA; Baseline-ZERO from Epicentre, Charlotte, USA; and Benzonase from Novagen, Darmstadt, Germany) and RNase (Promega, Madison, WI, USA) to digest unprotected nucleic acid at 37°C for 60 min (14). Total nucleic acids were then extracted using QIAamp MinElute Virus Spin Kit (Qiagen, HQ, Germany) according to the manufacturer's protocol. These nucleic acid pools containing DNA and RNA viral sequences were subjected to reverse transcription reactions with SuperScript III reverse transcriptase (Invitrogen, CA, USA) and 100 pmol of a random hexamer primer, followed by a single round of DNA synthesis using Klenow fragment polymerase (New England Biolabs, MA, USA). Thirty-three libraries were constructed using Nextera XT DNA Sample Preparation Kit (Illumina, CA, USA). Paired end reads of 250 bp generated by MiSeq were debarcoded using vendor software from Illumina for bioinformatics analysis. An in-house analysis pipeline running on a 32-node Linux cluster was used to process the data. Reads were considered duplicates if bases 5–55 were identical and only one random copy of duplicates was kept. Clonal reads were removed, and low sequencing quality tails were trimmed using Phred quality score 20 as the threshold. Adaptors were trimmed according to the default parameters of VecScreen, which is National Center for Biotechnology Information (NCBI) BLASTn with specialized parameters designed for adapter removal. The cleaned reads were *de novo* assembled within each barcode using the ENSEMBLE assembler (15). All generated contigs and singlets reads were then matched against a customized viral proteome database using BLASTx with an E-value cutoff of <10^−5^, where the virus BLASTx database was compiled using NCBI virus reference proteome (ftp://ftp.ncbi.nih.gov/refseq/release/viral/) to which was added viral proteins sequences from NCBI nr fasta file (based on annotation taxonomy in Virus Kingdom). Contigs without significant BLASTx similarity to viral proteome database are searched against viral protein families in vFam database (16) using HMMER3 to detect remote viral protein similarities (17–19). The BLASTx results generated by DIAMOND (DAA format) were imported into Megan6 for generation of rma6 format files which were further used for subsequent analysis including species rarefaction curve, species accumulation curve, and Co-occurrence plot.

### Statistical Analysis

All statistical analyses and normalization were performed using MEGAN6 and R version 4.0.3. Alpha-diversity and beta-diversity analysis was executed using the vegan package and a *P* < 0.05 was considered statistically significant, the ggplot2, and ggpubr packages were used for visual presentation. Permute, lattice, vegan, and ape packages were used to performed principal coordinate analysis (PCoA) based on Bray-Curtis dissimilarity (20). Statistical Analysis of Metagenomic Profiles (STAMP) was used to analyze the difference in viral communities composition between the AD patients group and the healthy controls group. The linear discriminant analysis effect size (LEfSe) was computed with alpha value lower than 0.05 and have an LDA score >3.0 (21).

### Quality Control

Standard precautions were used for all procedures to prevent cross-sample contamination and nucleic acid degradation. Aerosol filter pipet tips were used to reduce the possibility of sample cross contamination, and all the materials (including microcentrifuge tubes, pipet tips, etc.) which directly contacted with nucleic acid samples were RNase and DNase free. Nucleic acid samples were dissolved in DEPC treated water and RNase inhibitors were added. As a blank control, sterile ddH_2_O (Sagon, Shanghai, China) was simultaneously prepared and further processed in the same condition. During quality inspection using agarose gel electrophoresis and Agilent bioanalyzer 2100, no detectable DNA existed in the control library. During sequencing of Illumina MiSeq platform, the control library generated a small number of sequence reads. BLASTx searching based on the total reads in control library revealed no viral sequences.

### Data Availability

The raw sequence reads from the metagenomic libraries were deposited in the Short Read Archive (SRA) of the GenBank database with accession no. PRJNA666005.

## Results

### Demographic Characteristics of Healthy Controls and AD Patients

The study population included 21 AD patients and 12 healthy controls. The mean age of the AD patients and healthy controls was 10 and 10.5 years, respectively. There was no statistical difference in sex between AD patients group and healthy controls group (*P* > 0.05). The AD patients group and the healthy controls group also have no significant statistical significance in total, male or female age (*P* > 0.05) (Supplementary Table 1). The mean BSA (Body Surface Area) score of AD patients were 7.8 ± 3.5 (5.5–20.8). The mean EASI (Eczema area and severity index) score of AD patients were 6.5 ± 2.7 (3.8–17.4).

### Diversity and Richness of Gut Virome in AD Patients and Healthy Controls

**Figure 1:**
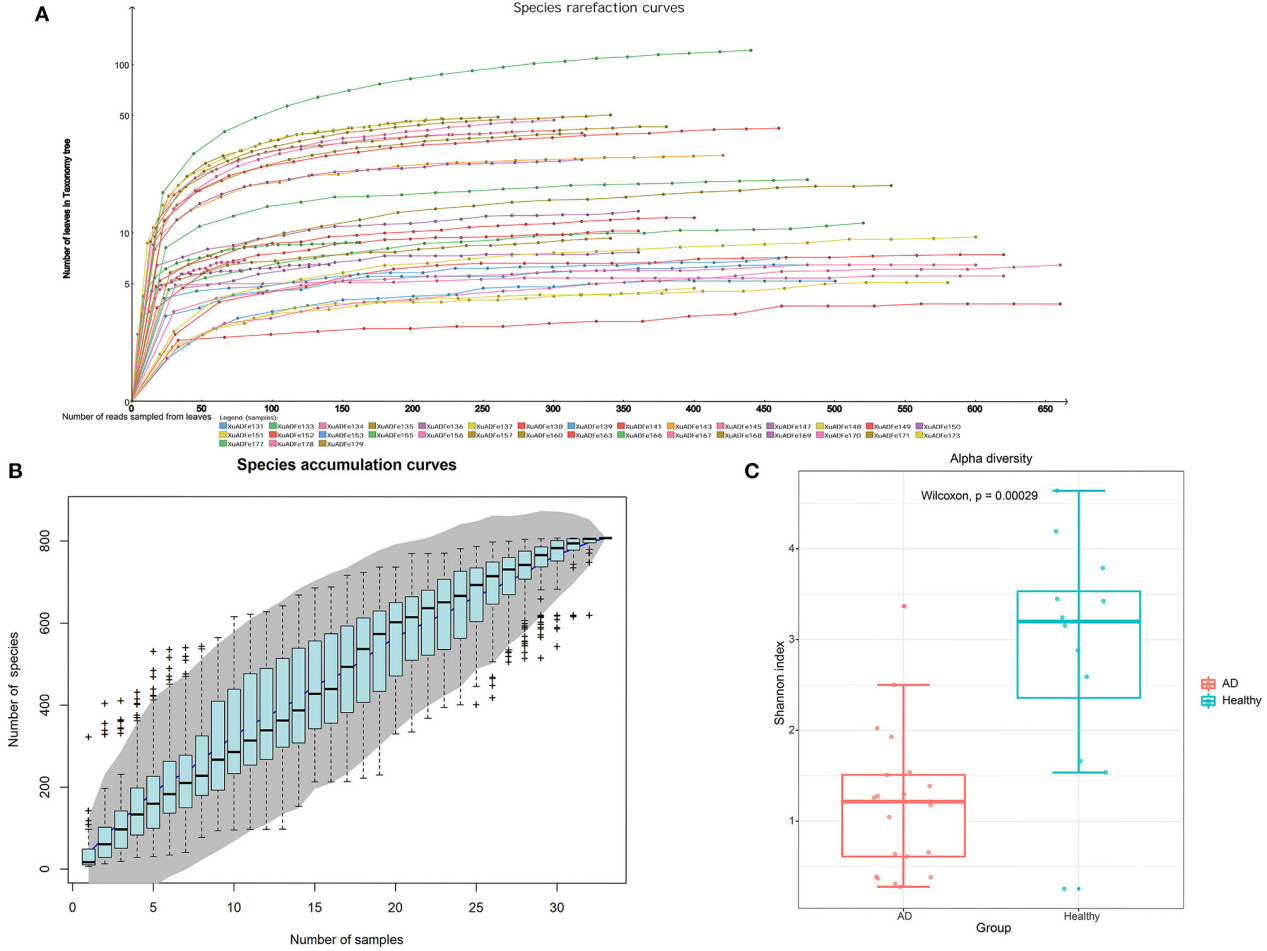
The richness and alpha diversity of viral species. **(A)** Species rarefaction curve drawn by Megan6 software after log scale transformation. Legends are displayed at the bottom of the figure, and each legend represents a library whose color corresponds to the color of the curve. **(B)** Based on the species accumulation curve of individual pool, the abscissa represents the number of sampled libraries, and the ordinate represents the cumulative number of species found. The gray shading indicates the 95% confidence interval. **(C)** Comparison of virus alpha diversity (Shannon index) between the AD patients group and the healthy controls group. The horizontal bars within boxes represent medians. The tops and bottoms of boxes represent the 75th and 25th percentiles, respectively. The upper and lower whiskers extend to data no more than 1.5× the interquartile range from the upper edge and lower edge of the box, respectively. The numbers of samples in this figure are as follows: AD patients (*n* = 21), healthy controls (*n* = 12).

After metagenomic sequencing, a total of 38,854,703 raw reads (13,047–4,357,473 per pool) were obtained from 33 libraries. Sequence reads were assembled *de novo* for each barcode and compared with the GenBank non-redundant protein database using BLASTx. In these libraries, 14,525,793 reads were associated with virus (721–1,617,209 per pool). The species rarefaction curve and species accumulation curve reflected the species richness of all collected samples (Figures 1A,B). With the increase of the number of reads sampled, the curve gradually transitioned to a plateau, which indicates that the sequencing depth in this research was large enough that more data would only reveal a small number of new species. It is estimated that the 33 libraries contain more than 800 viral species. Alpha diversity was used to clarify the difference in viral community composition between the AD patients group and the healthy controls group, and the Shannon index showed that the healthy controls group had significantly higher alpha diversity (*P* < 0.01, Wilcoxon test) (Figure 1C).

**Figure 2:**
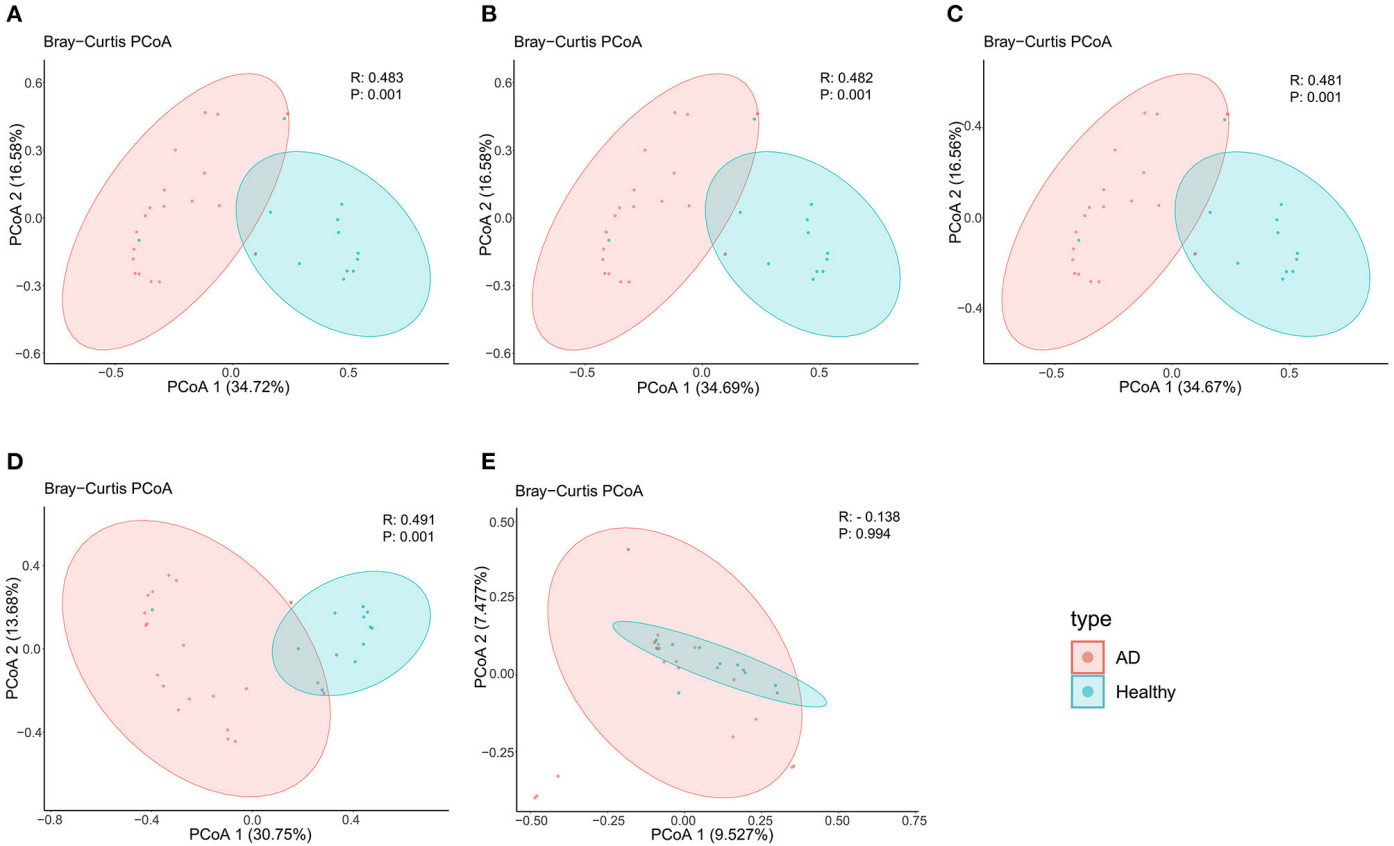
PCoA analysis of AD patients and healthy controls group at the **(A)** phylum, **(B)** class, **(C)** order, **(D)** family, and **(E)** genus levels.

In terms of beta diversity, unweighted UniFrac analysis suggested that the PCoA can distinguish the healthy controls group from the AD patients group at the phylum, class, order, and family levels, but cannot distinguish these two groups at the genus level, this may be due to the large and disordered data making the within-group differences greater than the between-group differences; Specifically, it may be limited by the metagenomics DIAMOND alignment method, and the same read was annotated into two or even more virus genera, which increased the false-positive rate of virus species counting, thus making the results biased (Figure 2).

### Viral Composition in the Feces

**Figure 3:**
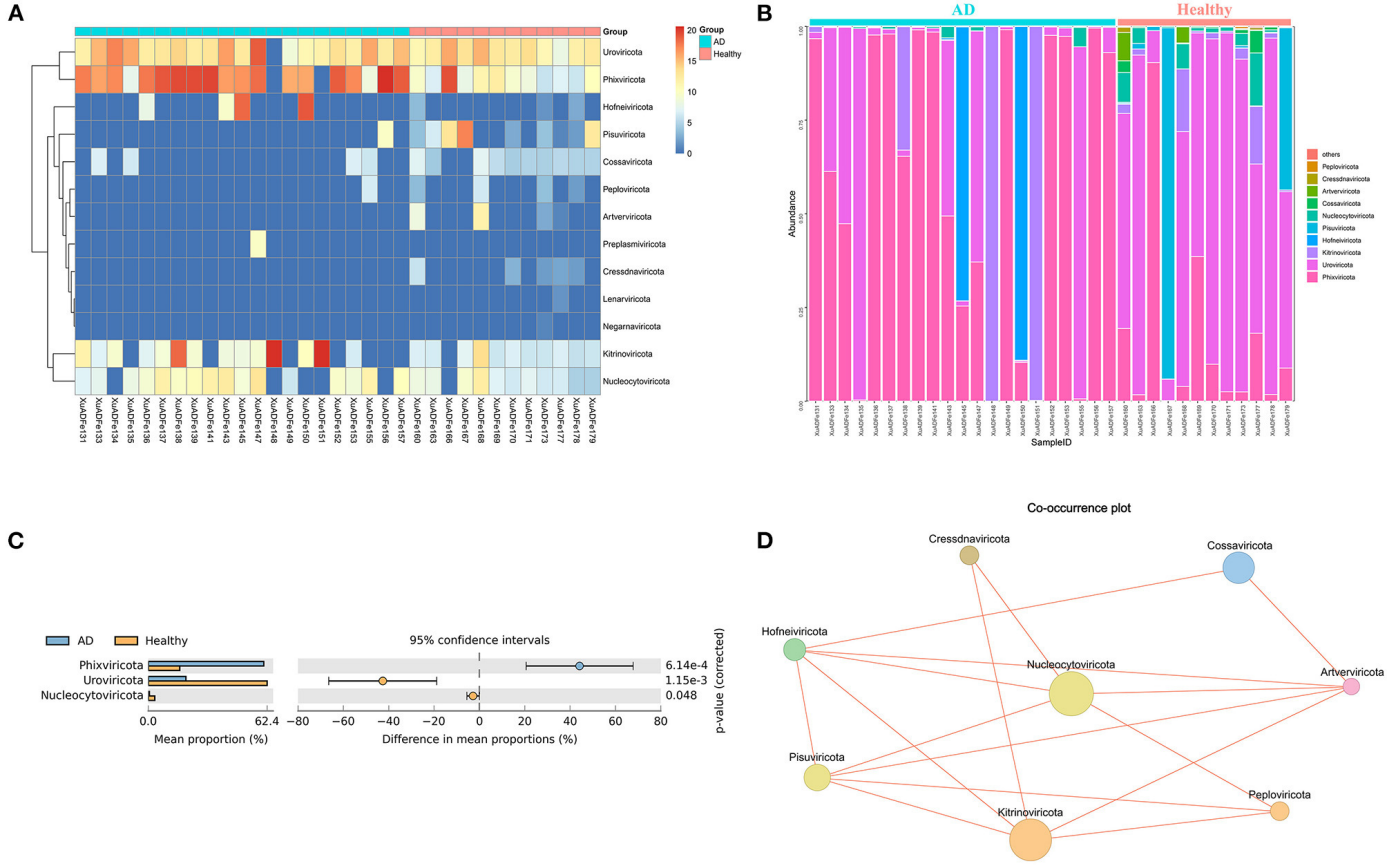
Analysis of differences in the composition of gut viral communities in AD patients and healthy controls group at the phylum level. **(A)** Clustering heatmap of representative virus families from 33 pools. The column name at the bottom of the figure indicates the pool number. The blue bar at the top of the figure represents the AD patients group, and the red bar represents the healthy controls group. The row name on the right side of the figure represents the name of the virus phylum. The number of reads is logarithmically converted with log10 as the base, and the legend is shown in the upper right corner. **(B)** Bar graph of viral community analysis of AD patients and healthy controls. **(C)** Analysis of differences between groups using STAMP. **(D)** Network analysis. Co-occurrence plot drawn with Megan6 software. Jaccard index was used to compare the similarity and difference between sample sets. The red line segment represents the anti-occurrence edge.

According to fecal metagenomic analysis, a total of 6 out of the 13 phyla showed different compositions (≥2-fold change) between healthy controls and AD patients (Supplementary Table 2). In these six phyla, *Pisuviricota* accounted for the highest proportion of viruses at 17.19% in the healthy group, followed by *Kitrinoviricota* (1.25%), *Nucleocytoviricota* (0.64%), and *Cossaviricota* (0.05%). On the other hand, *Kitrinoviricota* and *Hofneiviricota* were dominant in the AD patients group, accounting for 27.77 and 7.07% of viruses, respectively, and were 4,462.55 times and 22.14 times higher than those in healthy controls (Figures 3A,B). Statistical Analysis of Metagenomic Profiles analysis revealed that *Phixviricota* was the major factor in the difference between AD patients and healthy controls group (*P* < 0.01), followed by *Uroviricota* (*P* < 0.01) and *Nucleocytoviricota* (*P* < 0.05) (Figure 3C). The Co-occurrence plot showed that each virus phylum was negatively correlated with the other 2–5 phyla (Figure 3D).

**Figure 4:**
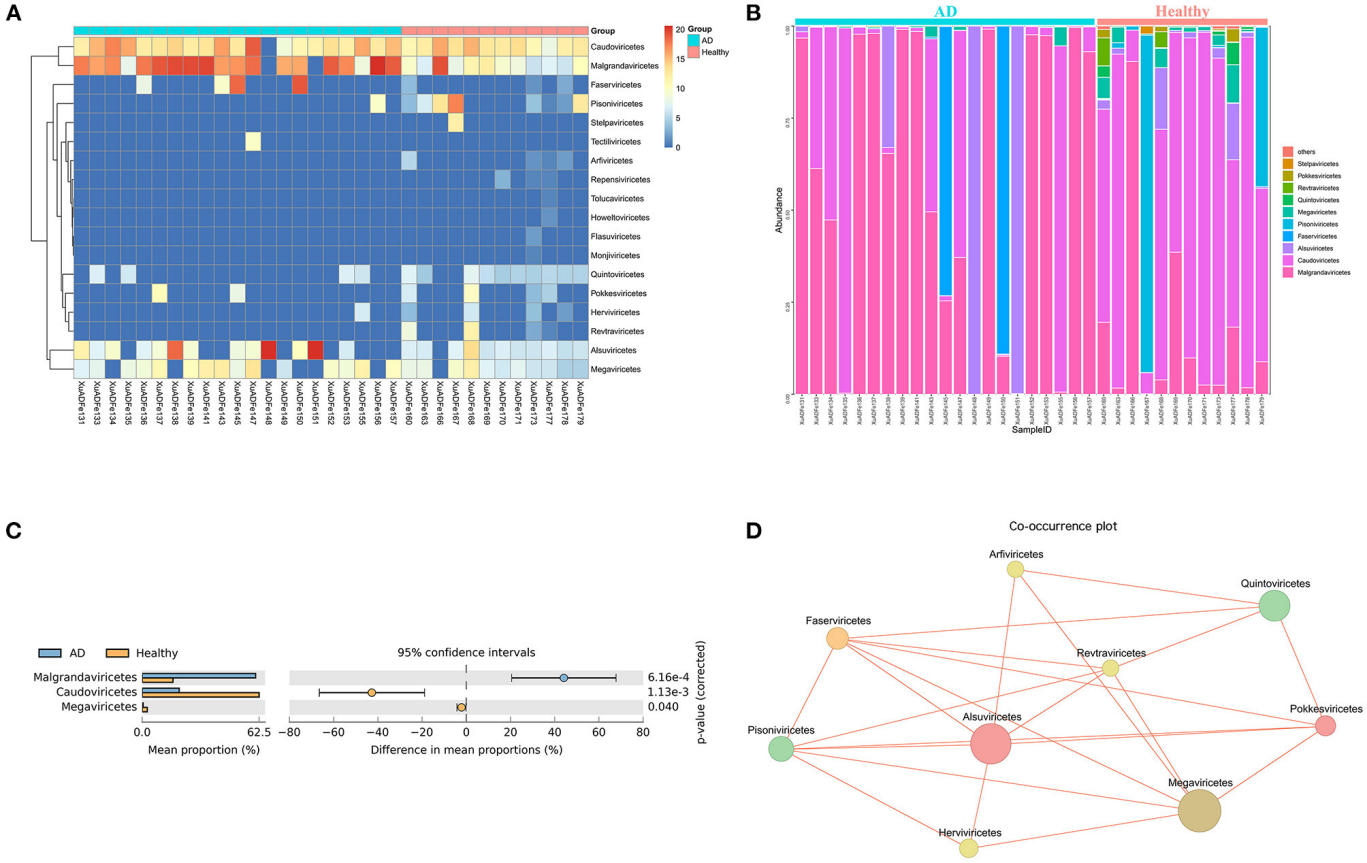
Analysis of differences in the composition of gut viral communities in AD patients and healthy controls group at the class level. **(A)** Clustering heatmap of representative virus families from 33 pools. The column name at the bottom of the figure indicates the pool number. The blue bar at the top of the figure represents the AD patients group, and the red bar represents the healthy controls group. The row name on the right side of the figure represents the name of the virus class. The number of reads is logarithmically converted with log10 as the base, and the legend is shown in the upper right corner. **(B)** Bar graph of viral community analysis of AD patients and healthy controls. **(C)** Analysis of differences between groups using STAMP. **(D)** Network analysis. Co-occurrence plot drawn with Megan6 software. Jaccard index was used to compare the similarity and difference between sample sets. The red line segment represents the anti-occurrence edge.

At the class level, 7 of the 18 classes have different compositions (≥2-fold change) between the two groups. The difference in the proportion of *Faserviricetes* between healthy controls and AD patients was the highest, about 4462.70 times. *Pisoniviricetes, Megaviricetes, Pokkesviricetes, Quintoviricetes*, and *Herviviricetes* in healthy controls were more than 2-fold change that of the AD patients group. In addition, *Alsuviricetes* accounted for 27.77% in the AD patients group and only 1.25% in the healthy controls (Figures 4A,B). According to the results of STAMP analysis, *Malgrandaviricetes, Caudoviricetes*, and *Megaviricetes* made the greatest contribution to the difference between these two groups (Figure 4C). The Co-occurrence plot displayed that of the nine classes, each class was negatively correlated with the other three to six classes (Figure 4D).

Simultaneously, among the seven orders with different compositions (≥2-fold change), *Picoravirales* was dominant in the healthy controls group, with 16.84% abundance, while *Martellivirales* was dominant in the AD patients group with 27.77% abundance. The amounts of *Piccovirales, Pimascovirales, Herpesvirales*, and *Chitovirales* in healthy controls were shown 7.41–17.25 times that of AD patients, while the amounts of *Tubulavirales* (7.08%) in AD patients was 4462.42 times of that in healthy controls. Among all 22 orders, *Petitvirales, Caudovirales, Imitervirales*, and *Pimascovirales* have significant contributions to the difference between the AD patients group and the healthy controls group. Based on co-occurrence plot, each of the 11 orders was negatively correlated with the other 1–8 orders (Supplementary Figure 1).

At the family level, there were six viral taxa over 0.1% in the AD patients group and 16 in the healthy controls group. Among the 12 families with different compositions (≥2-fold change), *Myoviridae* accounted for the highest proportion of the healthy controls group, reaching 1.60%, followed by *Virgaviridae* (1.26%), *Caliciviridae* (0.62%), *Drexlerviridae* (0.40%), and *Astroviridae* (0.37%), while *Virgaviridae* was dominant in the AD patients group with 27.84% abundance, and the proportion of *Inoviridae* in the AD patients was 4410.67 times that of the healthy controls group. Among all the 38 families detected, *Microviridae, Myoviridae, Mimiviridae*, and *Siphoviridae* were the dominant factors for the significant differences between the two groups. Different from the results of the above Co-occurrence plots, there was a positive correlation between *Iridoviridae* and *Retroviridae*, between *Circoviridae* and *Bromoviridae*, between *Mimiviridae* and *Podoviridae*, between *Herelleviridae* and *Ackermannviridae*, and between *Herelleviridae* and *Parvoviridae* (Supplementary Figure 2).

Finally, 55 and 15 viral genera accounted for over 0.1% of the total viral population in the healthy control group and the AD patients group, respectively. Among the 28 genera with different compositions (≥2-fold change), *Parechovirus, Tobamovirus, Enterovirus, Uetakevirus*, and *Mamastrovirus* were dominant in the healthy controls, particularly, *Parechovirus* accounted for 70.04% in the healthy controls. According to the results presented by STAMP, *Chlorovirus, Lubbockvirus, Mimivirus*, and *Coetzeevirus* made the greatest contribution to the difference between AD patients and healthy controls group. There also was a positive correlation between *Lederbergvirus* and *Hendrixvirus*, between *Cequinquevirus* and *Hendrixvirus*, between *Chlorovirus* and *Mimivirus*, and between *Cequinquevirus* and *Chlorovirus* (Supplementary Figure 3).

## Discussion

In westernized countries, the prevalence of AD has been on the rise, presumably due to the reduced exposure of the host immune system to beneficial microorganisms that accompanies the excessive hygiene of the modern lifestyle (5). The pathogenesis of AD is still unclear, its development involves genes, innate and adaptive immune responses, epidermal epithelial dysfunction, and multiple environmental factors (22, 23). It has been found that the gut microbiome can regulate the immune system by interacting with the host, thereby affecting distal immunity (such as the skin) and shaping the propensity of individuals to develop AD during infancy and early childhood and continue into adolescence and adulthood (4, 5, 24, 25). Although most reports describe the comparison of skin and gut bacterial composition between AD patients and healthy controls, the effects of gut virome on AD are rarely studied.

**Figure 5:**
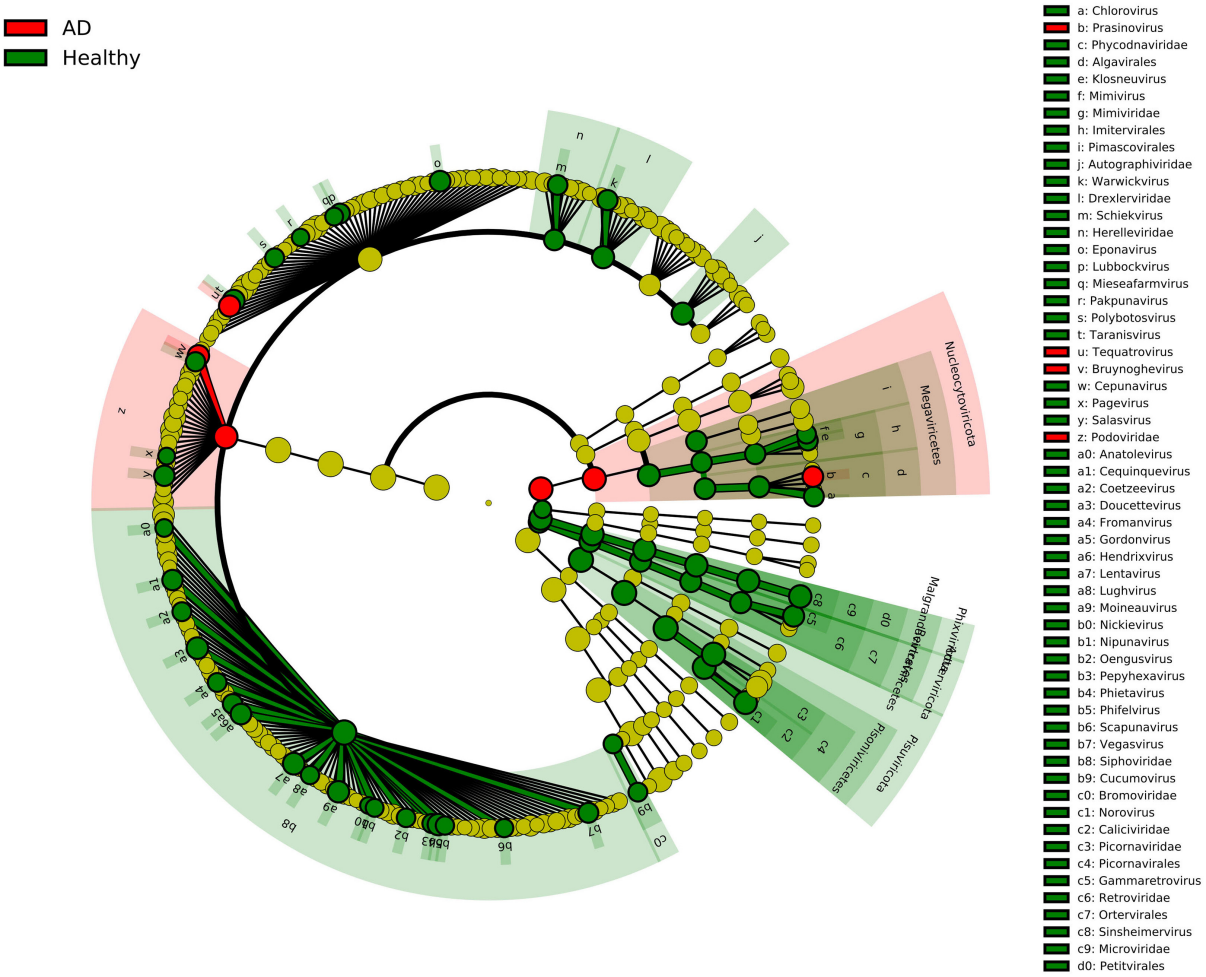
LEfSe analysis of viral community composition in AD patients group and healthy controls group. Cladogram for taxonomic representation of significant differences between AD patients group and healthy controls group. The colored nodes from inside to outside represent taxa from phylum to genus level. Only taxa with LDA values of 3.0 or higher are shown.

In this study, we recruited two groups of individuals with similar mean ages, including 21 AD patients and 12 healthy controls, and compared the composition of the gut viruses in the two groups at the five levels of the phylum, class, order, family, and genus. The rationality of the amount of sequenced data was inferred by drawing the species rarefaction curve and species accumulation curve. The number of reads at which saturation occurs in most libraries is about 15,000–80,000, and in a few libraries is about 2,000–10,000. It should be pointed out that the more than 800 different viral species contained in the 33 libraries are indeed a bit high (Figure 1B). This may be because the reads contained in the same viral genome have different best matches in BLASTx (*E*-value cutoff of <10^−5^), thereby falsely increasing the species of viruses. We combined analysis of species composition, α-diversity analysis, β-diversity analysis, and other analytical methods to evaluate the viral communities diversity of the data set. Overall, the significant difference in viral communities diversity between the AD patients and the healthy controls has been verified (Figure 1C). In the results section, we described viruses that have a 2-fold or more percentage change between the two groups in each taxonomy. For example, according to STAMP analysis, *Nucleocytoviricota*–*Megaviricetes*–*Imitervirales*–*Mimiviridae*–*Mimivirus* can distinguish the two groups at the level of the taxa to which it belongs. In fact, we have observed that as the classification level increases, the virus that helps distinguish AD patients from healthy controls may be the virus with the highest percentage in each group. The most typical ones were *Phixviricota–Malgrandaviricetes*–*Petitvirale–Microviridae*, which accounted for approximately 56.45 and 64.32% of AD patients and healthy controls at phylum, class, order, or family levels, respectively. In addition, *Uroviricota–Caudoviricetes–Caudovirales*, which seems to have no obvious statistical characteristic but was an important factor in distinguishing the composition of the two groups of viruses, accounting for about 8.43 and 16.20% in AD patients and healthy controls at phylum, class, order levels. Besides, the results of LEfSe analysis showed that at the level of phylum to genus, the dominant viruses in the gut of the healthy controls group were far more than that of the AD patients group (Figure 5). We may not be able to find a clear pattern to distinguish the characteristics of the viral communities composition in the gut of the two groups of individuals. Therefore, the difference in the composition of the two groups of viruses is still the result of multiple factors.

*Staphylococcus aureus* of the skin microbiome play key roles in the occurrence and development of AD and correlated with worsened disease severity. Previous studies have shown that whether the samples come from skin fluid or gut, the proportion of *S. aureus* in AD patients group was higher than that in healthy controls (26–31). It has been reported that *S. aureus* phages are assigned to three categories: *Podoviridae, Siphoviridae*, and *Myoviridae* (32). But in our study, *Podoviridae* was dominant in the AD group, while *Siphoviridae* and *Myoviridae* were dominant in the healthy group, which may be caused by the insufficient sample size or heterogeneity among sampled individuals. It is still unclear whether *S. aureus* phages may have some function in the gut, but we can partly speculate that *S. aureus* may have a reciprocal or symbiotic relationship with other bacteria, just as previous studies have confirmed that *Staphylococcus epidermidis* and *S. aureus* were prevalent at the same time when AD occurs (3, 33, 34).

It is worth noting that our research has some limitations. First, we did not perform group analysis according to the stage of disease progression of AD patients, which may result in different viral composition in different stages of AD. Second, the sample size used in this study was relatively small, so we still need to be cautious about the results obtained. Third, we did not perform metagenomic sequencing on the diseased skin area and the gut microbiome, and further research is needed to solve these limitations.

In conclusion, this study analyzed the composition and differences of gut viral communities between AD patients and healthy controls for the first time. Our study confirmed that the alpha diversity of the AD patients group was significantly lower than that of the healthy controls group, and the beta diversity of the two groups was significantly different from phylum to family level. The role of virome and microbiome in the gut on AD is still unknown, and the dominant viruses revealed by statistics may be affected by a variety of confounding factors. The differences in the gut viral communities between the healthy controls and AD patients may still be difficult to interpret and have unclear pattern, which requires further research to clarify these findings.

## Data Availability Statement

The datasets presented in this study can be found in online repositories. The names of the repository/repositories and accession number(s) can be found in the article/Supplementary Material.

## Ethics Statement

The studies involving human participants were reviewed and approved by Jiangsu University Ethics Committee. Written informed consent to participate in this study was provided by the participants' legal guardian/next of kin.

## Author Contributions

XL and HW contributed to the conception of the study. JZ, KJ, and LM contributed significantly to analysis and manuscript preparation. YW, SY, and XW performed the data analysis and wrote the manuscript QS, TZ, HX, and WZ helped perform the analysis with constructive discussions. All authors contributed to the article and approved the submitted version.

## Funding

This work was supported by National Key Research and Development Programs of China (No. 2017YFC1200201), the Maternal and Child Health Project of Jiangsu Province (No. F201717), Wuhan Medical Project (No. WX20B17), and the Doctor Project of Affiliated Hospital of Jiangsu University (No. jdfyrc2019003).

## Conflict of Interest

The authors declare that the research was conducted in the absence of any commercial or financial relationships that could be construed as a potential conflict of interest.

## Publisher's Note

All claims expressed in this article are solely those of the authors and do not necessarily represent those of their affiliated organizations, or those of the publisher, the editors and the reviewers. Any product that may be evaluated in this article, or claim that may be made by its manufacturer, is not guaranteed or endorsed by the publisher.

## Supplementary Material

Supplementary Figure 1 Analysis of differences in the composition of gut viral communities in AD patients and healthy controls group at the order level. **(A)** Clustering heatmap of representative virus families from 33 pools. The column name at the bottom of the figure indicates the pool number. The blue bar at the top of the figure represents the AD patients group, and the red bar represents the healthy controls group. The row name on the right side of the figure represents the name of the virus order. The number of reads is logarithmically converted with log10 as the base, and the legend is shown in the upper right corner. **(B)** Bar graph of viral community analysis of AD patients and healthy controls. **(C)** Analysis of differences between groups using STAMP. **(D)** Network analysis. Co-occurrence plot drawn with Megan6 software. Jaccard index was used to compare the similarity and difference between sample sets. The red line segment represents the anti-occurrence edge.

Supplementary Figure 2 Analysis of differences in the composition of gut viral communities in AD patients and healthy controls group at the family level. **(A)** Clustering heatmap of representative virus families from 33 pools. The column name at the bottom of the figure indicates the pool number. The blue bar at the top of the figure represents the AD patients group, and the red bar represents the healthy controls group. The row name on the right side of the figure represents the name of the virus family. The number of reads is logarithmically converted with log10 as the base, and the legend is shown in the upper right corner. **(B)** Bar graph of viral community analysis of AD patients and healthy controls. **(C)** Analysis of differences between groups using STAMP. **(D)** Network analysis. Co-occurrence plot drawn with Megan6 software. Jaccard index was used to compare the similarity and difference between sample sets. The green line segment represents the co-occurrence edge, and the red line segment represents the anti-occurrence edge.

Supplementary Figure 3 Analysis of differences in the composition of gut viral communities in AD patients and healthy controls group at the genus level. **(A)** Clustering heatmap of representative virus families from 33 pools. The column name at the bottom of the figure indicates the pool number. The blue bar at the top of the figure represents the AD patients group, and the red bar represents the healthy controls group. The row name on the right side of the figure represents the name of the virus genus. The number of reads is logarithmically converted with log10 as the base, and the legend is shown in the upper right corner. **(B)** Bar graph of viral community analysis of AD patients and healthy controls. **(C)** Analysis of differences between groups using STAMP. **(D)** Network analysis. Co-occurrence plot drawn with Megan6 software. Jaccard index was used to compare the similarity and difference between sample sets. The green line segment represents the co-occurrence edge, and the red line segment represents the anti-occurrence edge.

Supplementary Table 1 Demographic characteristics of healthy controls and AD patients. Data are expressed as mean ± standard deviation.

Supplementary Table 2 The summary of the percentage information of viruses under different categories.

## Abbreviations

BLAST: basic local alignment search tool
AD: atopic dermatitis
NCBI: National Center for Biotechnology Information.
