# Supplementary figures and images for: Comparison of Gut Viral Communities in Atopic Dermatitis and Healthy Children
Source: Front Med (Lausanne). 2022 Feb 21;9:835467. doi: 10.3389/fmed.2022.835467 (PMC8899399; doi:10.3389/fmed.2022.835467)

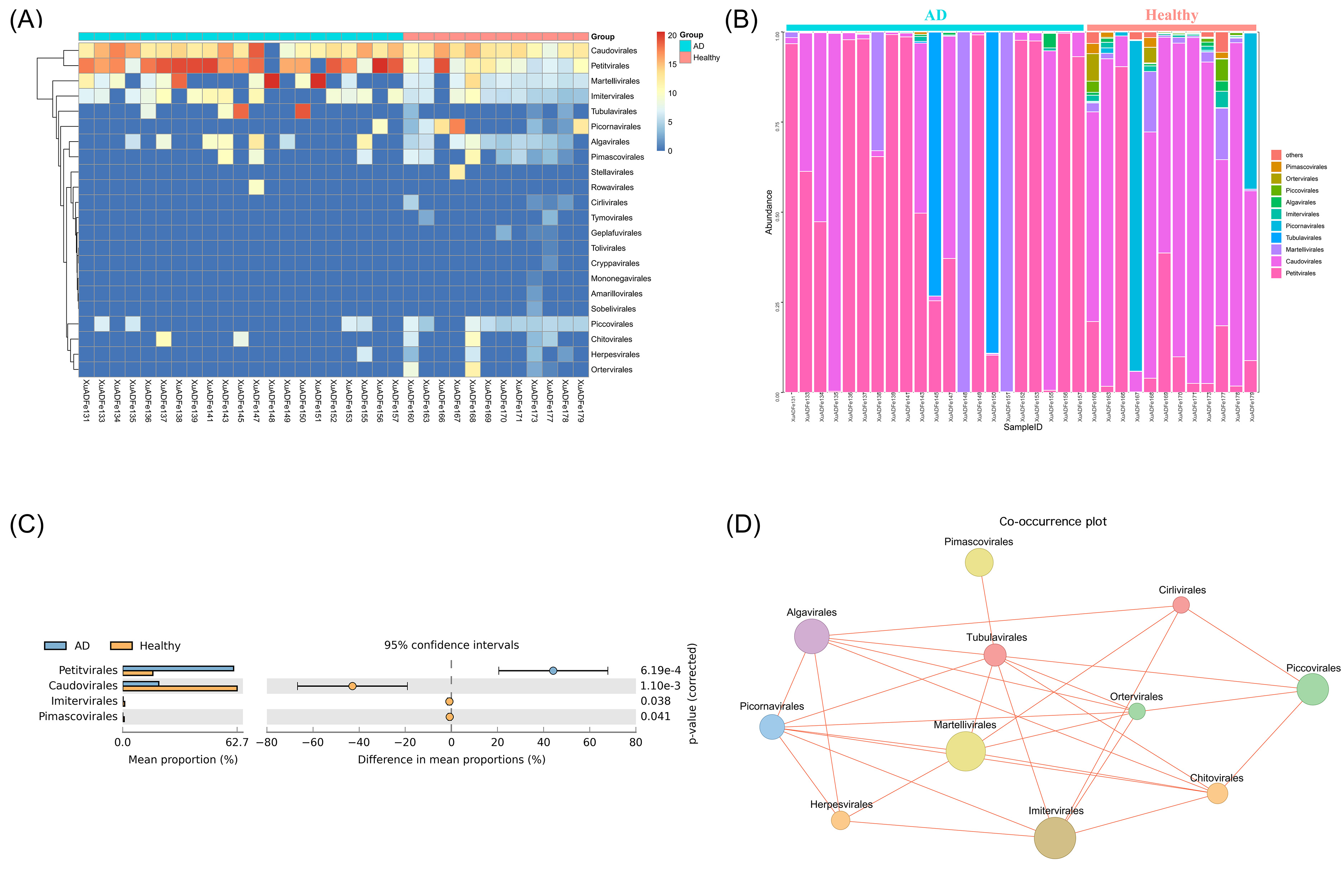

Supplement: Supplementary Figure 1 — Analysis of differences in the composition of gut viral communities in AD patients and healthy controls group at the order level. (A) Clustering heatmap of representative virus families from 33 pools. The column name at the bottom of the figure indicates the pool number. The blue bar at the top of the figure represents the AD patients group, and the red bar represents the healthy controls group. The row name on the right side of the figure represents the name of the virus order. The number of reads is logarithmically converted with log10 as the base, and the legend is shown in the upper right corner. (B) Bar graph of viral community analysis of AD patients and healthy controls. (C) Analysis of differences between groups using STAMP. (D) Network analysis. Co-occurrence plot drawn with Megan6 software. Jaccard index was used to compare the similarity and difference between sample sets. The red line segment represents the anti-occurrence edge. [file Image_1.JPEG]

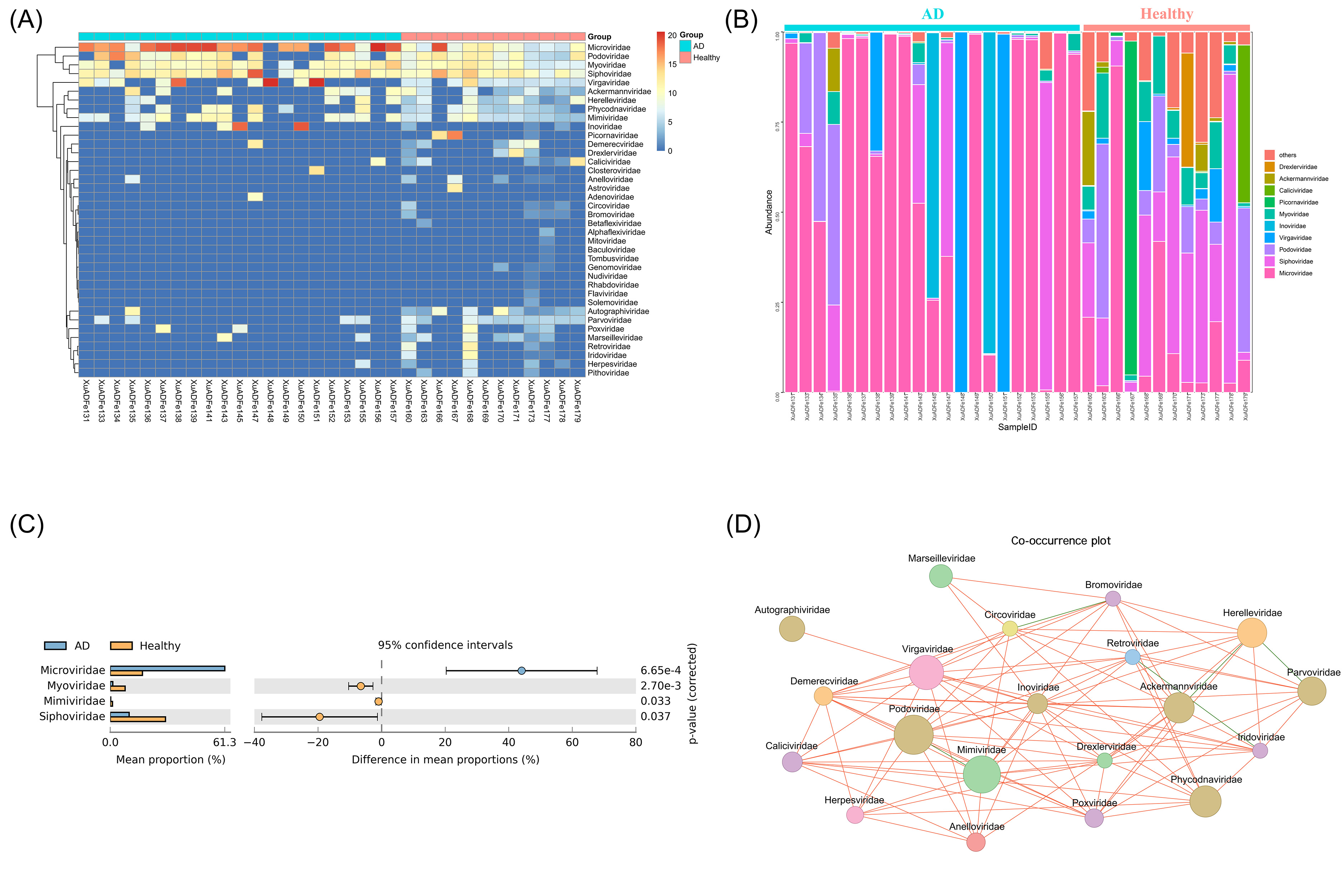

Supplement: Supplementary Figure 2 — Analysis of differences in the composition of gut viral communities in AD patients and healthy controls group at the family level. (A) Clustering heatmap of representative virus families from 33 pools. The column name at the bottom of the figure indicates the pool number. The blue bar at the top of the figure represents the AD patients group, and the red bar represents the healthy controls group. The row name on the right side of the figure represents the name of the virus family. The number of reads is logarithmically converted with log10 as the base, and the legend is shown in the upper right corner. (B) Bar graph of viral community analysis of AD patients and healthy controls. (C) Analysis of differences between groups using STAMP. (D) Network analysis. Co-occurrence plot drawn with Megan6 software. Jaccard index was used to compare the similarity and difference between sample sets. The green line segment represents the co-occurrence edge, and the red line segment represents the anti-occurrence edge. [file Image_2.JPEG]

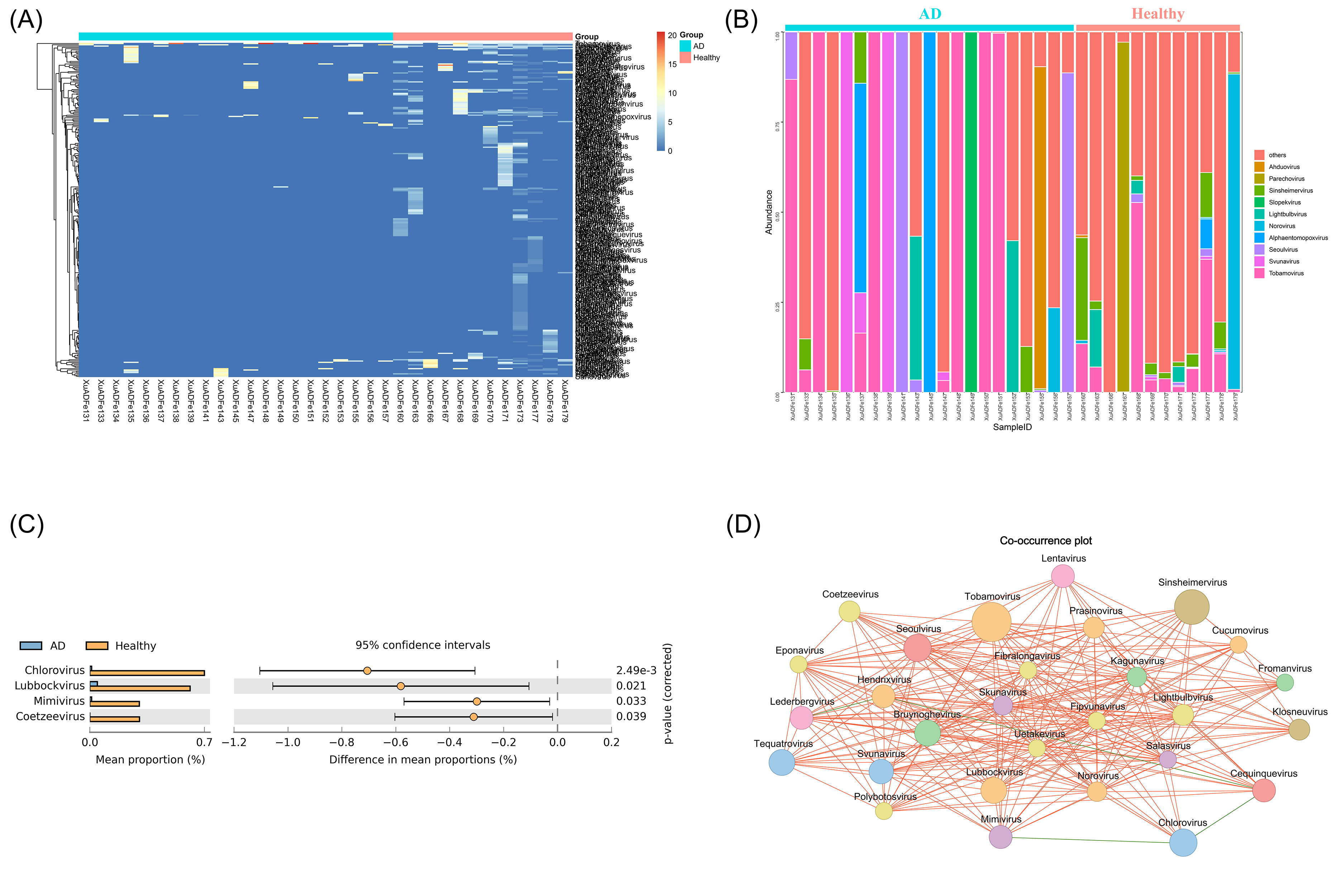

Supplement: Supplementary Figure 3 — Analysis of differences in the composition of gut viral communities in AD patients and healthy controls group at the genus level. (A) Clustering heatmap of representative virus families from 33 pools. The column name at the bottom of the figure indicates the pool number. The blue bar at the top of the figure represents the AD patients group, and the red bar represents the healthy controls group. The row name on the right side of the figure represents the name of the virus genus. The number of reads is logarithmically converted with log10 as the base, and the legend is shown in the upper right corner. (B) Bar graph of viral community analysis of AD patients and healthy controls. (C) Analysis of differences between groups using STAMP. (D) Network analysis. Co-occurrence plot drawn with Megan6 software. Jaccard index was used to compare the similarity and difference between sample sets. The green line segment represents the co-occurrence edge, and the red line segment represents the anti-occurrence edge. [file Image_3.JPEG]
