# Supplementary material for: Comparison of Gut Viral Communities in Atopic Dermatitis and Healthy Children
Source: Front Med (Lausanne). 2022 Feb 21;9:835467. doi: 10.3389/fmed.2022.835467 (PMC8899399; doi:10.3389/fmed.2022.835467)
Supplement: Supplementary Table 1 — Demographic characteristics of healthy controls and AD patients. Data are expressed as mean ± standard deviation. [file Table_1.pdf]

Variables	Control (n=12)	AD (n=21)	*P* value
Sex (Male/Female)	7/5	15/6	0.471
Age (years)			
Total	10.0±1.3	10.5±1.2	0.297
Male	10.6±0.9	11.0±1.0	0.477
Female	9.1±1.2	9.3±0.6	0.811
